# Validation of a portable, waterproof blood pH analyser for elasmobranchs

**DOI:** 10.1093/conphys/cox012

**Published:** 2017-02-27

**Authors:** Brendan Talwar, Ian A. Bouyoucos, Oliver Shipley, Jodie L. Rummer, John W. Mandelman, Edward J. Brooks, R. Dean Grubbs

**Affiliations:** 1 Coastal and Marine Laboratory, Florida State University, St. Teresa, FL 32358, USA; 2 Department of Natural Resources and Environmental Sciences, University of Illinois at Urbana-Champaign, Urbana, IL 61801, USA; 3 ARC Centre of Excellence for Coral Reef Studies, James Cook University, Townsville, Queensland 4811, Australia; 4 School of Marine and Atmospheric Sciences, Stony Brook University, Stony Brook, NY 11790, USA; 5 John H. Prescott Marine Laboratory, New England Aquarium, Boston, MA 02110, USA; 6 Shark Research and Conservation Program, Cape Eleuthera Institute, Rock Sound, The Bahamas

**Keywords:** Blood pH, i-STAT, *Negaprion brevirostris*, point-of-care device, *Squalus cubensis*, stress

## Abstract

Quantifying changes in blood chemistry in elasmobranchs can provide insights into the physiological insults caused by anthropogenic stress, and can ultimately inform conservation and management strategies. Current methods for analysing elasmobranch blood chemistry in the field are often costly and logistically challenging. We compared blood pH values measured using a portable, waterproof pH meter (Hanna Instruments HI 99161) with blood pH values measured by an i-STAT system (CG4+ cartridges), which was previously validated for teleost and elasmobranch fishes, to gauge the accuracy of the pH meter in determining whole blood pH for the Cuban dogfish (*Squalus cubensis*) and lemon shark (*Negaprion brevirostris*). There was a significant linear relationship between values derived *via* the pH meter and the i-STAT for both species across a wide range of pH values and temperatures (Cuban dogfish: 6.8–7.1 pH 24–30°C; lemon sharks: 7.0–7.45 pH 25–31°C). The relative error in the pH meter's measurements was ~±2.7%. Using this device with appropriate correction factors and consideration of calibration temperatures can result in both a rapid and accurate assessment of whole blood pH, at least for the two elasmobranch species examined here. Additional species should be examined in the future across a wide range of temperatures to determine whether correction factors are universal.

## Teaser

Analysis of blood chemistry, including whole blood pH, can provide a valuable metric of physiological disturbance in fishes exposed to anthropogenic stress. Here, we demonstrate the utility of a cost-effective, portable, waterproof pH meter in measuring lemon shark and Cuban dogfish whole blood pH.

## Introduction

Point-of-care blood analysers are commonly used as a non-lethal tool to evaluate the health and condition of animals in both veterinary and research settings (Thrall *et al*., 2012; Stoot *et al*., 2014). Analysing blood chemistry can improve our understanding of animal–environment interactions and provide physiological thresholds that may be critical in overcoming conservation challenges (Cooke and O'Connor, 2010; Stoot *et al*., 2014). Blood chemistry has proven particularly useful when quantifying the physiological effects of anthropogenic stress on fish (e.g. Cooke *et al*., 2005; Arlinghaus et al., 2009; Foss *et al*., 2012), including the effects of commercial (Hyatt *et al*., 2012), recreational (Bower *et al*., 2016a, b) and experimental (Brooks *et al*., 2012; Gallagher *et al*., 2014) capture, rates of recovery after catch and release (Kneebone *et al*., 2013), and stress associated with changes in water conditions (Shultz *et al*., 2014).

In capture and release scenarios, quantifying the physiological stress response is of interest in order to understand inter-specific differences in capture-induced mortality and post-release survival (Skomal and Mandelman, 2012), particularly for species subject to frequent bycatch interactions, such as many elasmobranchs (Oliver *et al*., 2015). Typically, fisheries capture induces a physiological stress response in sharks (and teleost fishes) that is characterized by varying degrees of metabolic and/or respiratory acidoses, the total magnitude of which can be quantified by measuring blood pH (Cliff and Thurman, 1984; Hoffmayer and Parsons, 2001; Mandelman and Skomal, 2009; Brooks *et al*., 2012). Significantly depressed blood pH can also be correlated with mortality (Cliff and Thurman, 1984; Skomal, 2006; Skomal and Mandelman, 2012), making it a valuable and commonly used metric to assess stress in field studies on elasmobranchs (e.g. Mandelman and Skomal, 2009; Brooks *et al*., 2012; Frick *et al*., 2012; Hyatt *et al*., 2012).

Measuring the pH of blood drawn from a shark in a field setting is often accomplished using the i-STAT system (Abbot Point of Care Inc., Princeton, NJ, USA) (see Stoot *et al*., 2014 for examples), a point-of-care analyser designed for the clinical analysis of blood at 37°C. While the i-STAT system has been validated for use in both teleosts and sharks (e.g. juvenile sandbar sharks *Carcharhinus plumbeus*, Gallagher *et al*., 2010; Harter *et al*., 2015; rainbow trout *Oncorhynchus mykiss*, Harter *et al*., 2014; adult dusky smoothhound *Mustelus canis*, Gallagher *et al*., 2010), analysing fish blood often requires a change in sample temperature that can alter blood pH (Brill *et al*., 1992; Gallagher *et al*., 2010). As such, reliable blood pH values are ideally derived from species-specific temperature correction factors (Gallagher *et al*., 2010) and the i-STAT's internal temperature correction formula (Harter *et al*., 2015). Unfortunately, only one equation for an elasmobranch is available (nursehound, *Scyliorhinus stellaris*; Mandelman and Skomal, 2009) although temperature correction is of extreme importance when sample temperature dependency differs from that of human blood (Malte *et al*., 2014). Further, the i-STAT system is costly and wasteful due to the expense of the unit itself as well as the single-use cartridges it requires (~$16 USD each) where user errors are common (34.5% of analyses yield partial results; Harter *et al*., 2015). Using the i-STAT in field settings is also challenging, as the unit is not waterproof and only operates within a narrow temperature range (18–30°C). There is a clear need to investigate the use of blood analysers that can function under a range of field conditions (e.g. in the tropics) and at reduced cost, yet no studies have validated the use of i-STAT alternatives for measuring shark blood pH.

The Hanna Instruments HI 99161 pH meter (Hanna Instruments, Woonsocket, RI, USA; ‘pH meter’) is one such alternative used in previous field studies on teleost fishes (Brownscombe *et al*., 2014, 2015; Bower *et al*., 2016a, b). The pH meter is a portable, waterproof, standalone unit designed for measuring the pH of meat and dairy products, which provides whole blood pH as a function of sample temperature that is measured with a built-in temperature sensor. To evaluate its application, we compared blood pH measurements between the i-STAT and the pH meter across a range of values and temperatures in two species of elasmobranchs: the lemon shark (*Negaprion brevirostris*), likely the most-studied, commercially important, coastal shark species to date (Dibattista *et al*., 2007; Ashe *et al*., 2015) and the Cuban dogfish (*Squalus cubensis*), a small-bodied deepwater shark regularly caught as bycatch in the northern Gulf of Mexico (Hale *et al*. 2010; Jones *et al*., 2013). Further, we examined the relative error of pH meter measurements when compared with the i-STAT system's results for both species. The major outcome of this study is a correction factor for each species that can be used for correcting blood pH values derived using the pH meter to values that are in line with what would be derived from an i-STAT upon temperature compensation, and, indirectly, conventional temperature-controlled laboratory pH electrodes (Harter *et al*., 2015).

## Materials and methods

### Animal ethics, collection and husbandry

Research was carried out under the CEI research permit numbers MAF/FIS/17 and MAF/FIS/34 issued by the Bahamas Department of Marine Resources in accordance with the University of Illinois Institutional Animal Care and Use Committee (ACUC) Protocol #15124, the Florida State University ACUC Protocol #1412, and CEI animal care protocols developed within the guidelines of the Association for the Study of Animal Behavior and the Animal Behavior Society (Rollin and Kessel, 1998). Permission to capture sharks within the Bahamas Shark Sanctuary was established in accordance with Bahamas Department of Marine Resources Form 20A, Regulation 36D (3), permitting fishing, possession and exportation of sharks or shark tissue.

Field work was conducted July 2014–April 2016 in northeastern Exuma Sound, ~2.5 km west of Powell Point (24.541°N, 76.121°W), and in three adjacent tidal mangrove creeks (Kemps, Broad and Page Creeks) in Cape Eleuthera, The Bahamas. Cuban dogfish were captured using standard demersal longlines set at 450–800 m in Exuma Sound. Surface water temperatures ranged from 24.9°C to 30.5°C over this period while the mean capture temperature at depth was 12.0 ± 1.8°C [±standard deviation (SD)]. Longlines were hauled to the surface using a commercially available electric pot hauler (Waterman Industries of Florida, Inc., Odessa, FL, USA) at a rate of 0.3 m/s. Lemon sharks were captured in tidal creeks by rod and reel, seines, or longlines during either winter months (water temperature: 26.3 ± 1.3°C; mean ± SD) or summer months (water temperature: 29.1 ± 1.6°C; mean ± SD) before being transferred to the Cape Eleuthera Institute's (CEI) wet laboratory.

Lemon sharks were maintained in 13 000 litre (3.7 m diameter by 1.2 m depth) flow-through holding tanks supplied with clean seawater from an offshore pump in a partially covered facility that exposed sharks to ambient water temperature (winter: 24.3–27.8°C; summer: 25.6–31.3°C) and a natural photoperiod. Sharks were fed commercially available frozen Spanish sardines (*Sardinella aurita*; roughly 6% of body weight daily), with the exception of a 48-h fast prior to experimentation. All sharks were typically released to their capture site after 2, but no >4, weeks in captivity.

### Blood sampling

The pH meter was calibrated when readings differed from calibration solution standards (usually weekly). Calibrations occurred in CEI's shaded, indoor laboratory at the same air temperatures that all blood samples were analysed (lab space was not temperature controlled and hovered around 21–26°C) and involved a two-point calibration in 7.01 (at 25°C) buffer solution (HI 50007; Hanna Instruments) and 4.01 (at 25°C) buffer solution (HI 50004; Hanna Instruments) following instructions in the pH meter's user manual.

Cuban dogfish were sampled immediately upon contact after longline retrieval after soak times ranging from 121 to 314 min, while lemon sharks were individually habituated to one tank for 24 h prior to sampling. To achieve variable blood pH measurements, lemon sharks were exposed to varying exercise regimes including hooking on experimental longline gangions and exhaustive chasing procedures as part of unrelated, ongoing investigations. Exhaustive chasing is thought to elicit similar physiological responses in fishes as hook-and-line capture (Kieffer, 2000), but ultimately both techniques result in reductions in blood pH associated with a secondary stress response.

Sharks were placed into tonic immobility while submerged, and blood (~1–3 ml) was drawn by caudal venipuncture using a 25.4-mm, 22-gauge needle and either a 3-ml or a 5-ml heparinized syringe. Roughly 95 μl of blood was then inserted into an i-STAT CG4+ cartridge to measure blood pH with an i-STAT blood gas analyser (Heska Corporation, Fort Collins, CO, USA) thermoset to 37°C (Mandelman and Skomal, 2009; Gallagher *et al*., 2010; Harter *et al*., 2015). Cartridges were stored inside their original packaging at 4**°**C, but allowed to sit at room temperature for 5 min prior to use. Simultaneously, 1 ml of blood was transferred to a 1.5-ml Eppendorf tube housed inside an insulating foam sleeve (to reduce any possible differences between *in vivo* animal temperature and blood temperature at the time of analysis) and analysed using the pH meter to determine both blood temperature and pH. Analyses typically occurred within 2 min of caudal venipuncture; values that were recorded after 5 min were discarded.

### Data analysis

Measurements derived from the i-STAT were temperature corrected (according to the i-STAT's internal temperature correction function, where blood temperature determined by the pH meter was substituted in for patient temperature), while pH meter values were automatically temperature corrected to that of the blood sample using a built-in temperature correction formula. Note, the pH meter does not change the temperature of the sample, instead it measures sample temperature and uses that to account for temperature-induced errors associated with the built-in electrode. Then, i-STAT measurements were corrected again using the equation reported in Harter *et al*. (2015) to match laboratory-derived pH values. All data were examined for normality and outliers using diagnostic plots. The relationships between normally distributed variables were examined using linear regression analyses. Linear models generated for different sampling periods were compared using an analysis of covariance (ANCOVA). The relative error between the pH meter and i-STAT was calculated using the following equation: δpH (%) = (pH meter pH − i-STAT pH)/i-STAT pH × 100. Relative error measurements were then regressed with i-STAT values (see Harter *et al*., 2015). All analyses were performed using JMP 7.0.1 (SAS Institute, Cary, NC, USA) and the level of significance for the aforementioned tests was *α* < 0.05.

## Results

### Cuban dogfish

**Figure 1:**
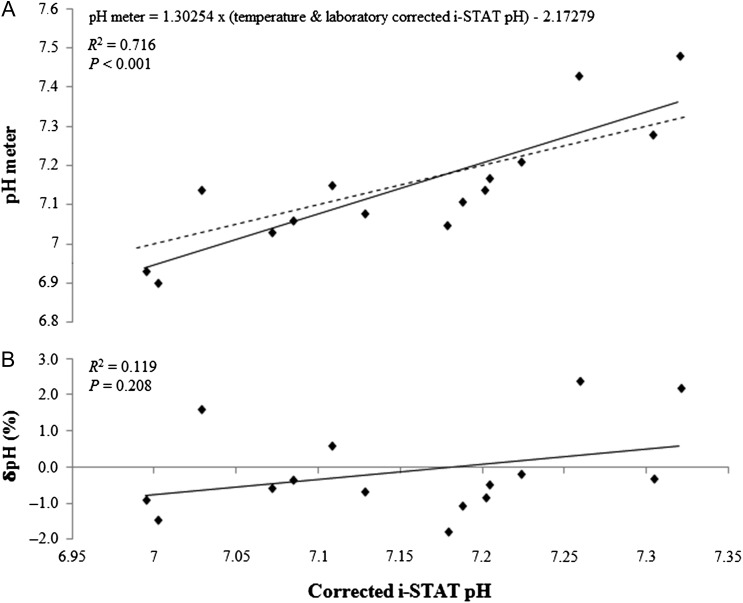
(**A**) Cuban dogfish whole blood pH measured with the Hanna Instruments HI 99161 pH meter vs. pH measured with the i-STAT device and corrected with laboratory (Harter *et al*., 2015) and internal temperature correction formulas. (**B**) The relative error of the pH meter measurements, δpH (%) [(pH meter pH − i-STAT pH)/i-STAT pH × 100], vs. corrected i-STAT pH measurements.

Linear regression suggested a significant relationship between pH meter measurements and i-STAT measurements of blood pH (*P* < 0.001, *R*^2^ = 0.716; Fig. 1A). This relationship can be predicted using the following equation:

(1) $$\begin{array}{c} \text{pH meter}\;=\;1.30254\times (\text{temperature}\;\text{and laboratory}\\ \;\;\text{corrected i STAT}\;\mathrm{pH})-2.17279 \end{array}$$

There was no significant relationship between δpH (%) and i-STAT pH (*P* = 0.208, *R*^2^ = 0.119; Fig. 1B).

### Lemon shark

**Figure 2:**
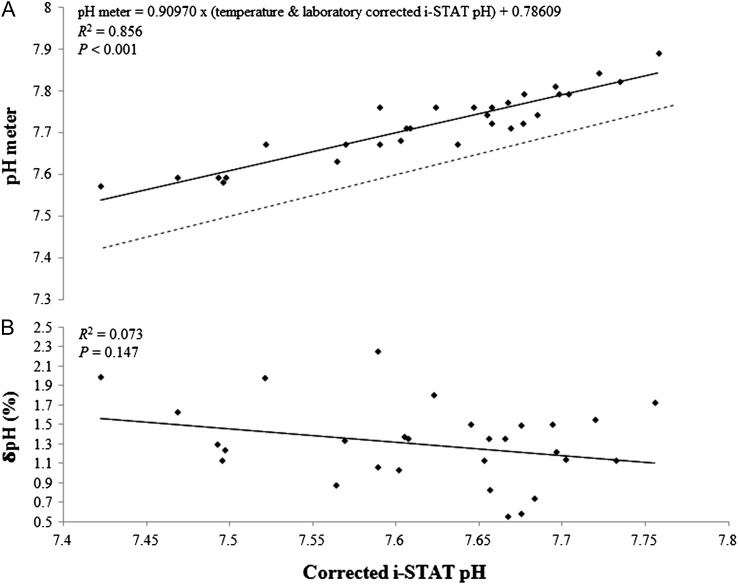
(**A**) Lemon shark whole blood pH measured during the colder sampling period (water temperature: 26.3 ± 1.3°C; mean ± SD) with the Hanna Instruments HI 99161 pH meter vs. pH measured with the i-STAT device and corrected with laboratory (Harter *et al*., 2015) and internal temperature correction formulas. (**B**) The relative error of the pH meter measurements, δpH (%) [(pH meter pH − iSTAT pH)/iSTAT pH × 100], vs. corrected i-STAT pH measurements.

There was a significant relationship between pH meter measurements and i-STAT measurements of blood pH during the colder sampling period (*P* < 0.001, *R*^2^ = 0.856; Fig. 2A). This relationship can be predicted using the following equation:

(2) $$\begin{array}{c} \text{pH meter}\;=\;0.90970\;\times \;(\text{temperature and laboratory}\\ \;\text{corrected i-STAT pH})+\text{0}.78609 \end{array}$$

There was no significant relationship between δpH (%) and i-STAT pH (*P* = 0.147, *R*^2^ = 0.073; Fig. 2B).

**Figure 3:**
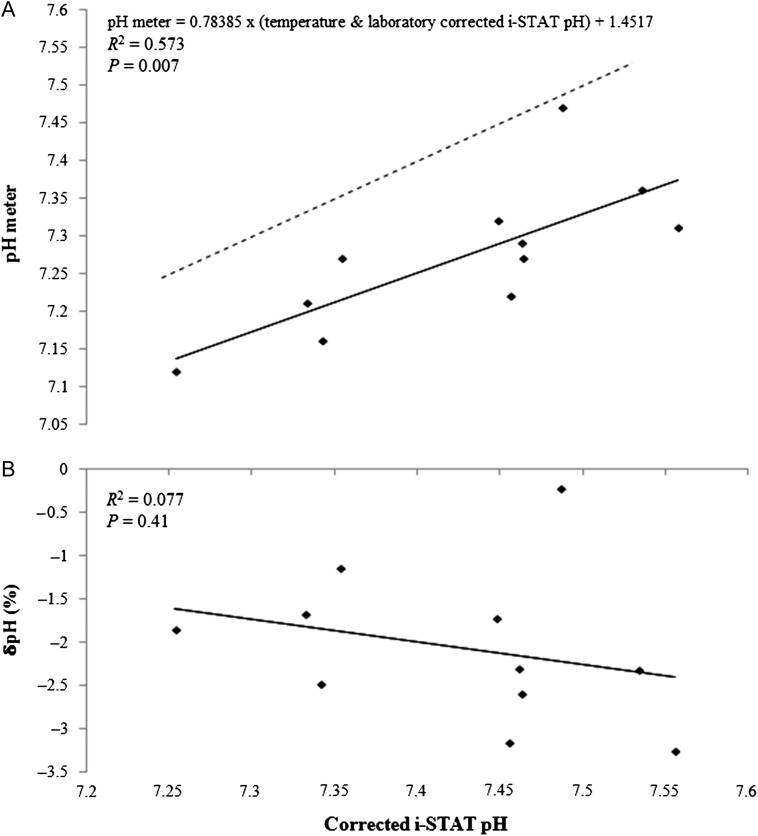
(**A**) Lemon shark whole blood pH measured during the warmer sampling period (water temperature: 29.1 ± 1.6°C; mean ± SD) with the Hanna Instruments HI 99161 pH meter vs. pH measured with the i-STAT device and corrected with laboratory (Harter *et al*., 2015) and internal temperature correction formulas. (**B**) The relative error of the pH meter measurements, δpH (%) [(pH meter pH − iSTAT pH)/iSTAT pH × 100], vs. corrected i-STAT pH measurements.

There was also a significant relationship between pH meter measurements and i-STAT measurements of blood pH during the warmer sampling period (*P* = 0.007, *R*^2^ = 0.573; Fig. 3A). This relationship can be predicted using the following equation:

(3) $$\begin{array}{c} \text{pH meter}=\;0.78385\times \left(\text{temperature and laboratory corrected i-STAT pH}\right)+\text{1}.45170 \end{array}$$

There was no significant relationship between δpH (%) and i-STAT pH (*P* = 0.41, *R*^2^ = 0.077; Fig. 3B), although the δpH (%) was more positive for these samples than for those taken during winter months. There was also no significant difference between the linear models generated for winter and summer sampling periods for this species (ANCOVA interaction term > 0.05).

## Discussion

We established significant linear relationships between the values derived *via* the pH meter and the i-STAT device for both Cuban dogfish and lemon shark whole blood across a range of pH values and, for the lemon shark, over two seasonal temperature ranges. The pH meter's relative measurement error (δpH) for Cuban dogfish whole blood pH was between −1.8 and 2.4% (0.01–0.17 pH units), while for lemon sharks δpH ranged from −3.25 to −0.22% (0.03–0.17 pH units) during the colder months and from 0.56 to 2.25% (0.02–0.25 pH units) during the warmer months. These measurement errors are similar to those reported for the i-STAT system by Harrenstien *et al*. (2005), Gallagher *et al*. (2010) and Harter *et al*. (2015) when tested against more conventional laboratory blood analysers.

Given the significant linear relationships established here, pH meter values can ideally be converted to their laboratory- and temperature-corrected equivalents, or, alternatively, can be reported without applying correction factors to compare the relative stress of conspecifics after exposure to a stressor, which is widely accepted in field-based stress physiology studies on fishes using both the pH meter (Brownscombe *et al*., 2014, 2015; Bower *et al*., 2016a, b) and the i-STAT system (Brill *et al*., 2008; Mandelman and Skomal, 2009; Brooks *et al*., 2011; Frick *et al*., 2012; Hyatt *et al*., 2012).

Some caution should be taken, however, as there was higher relative measurement error associated with the warmer sampling period for lemon shark whole blood pH, which could be driven by confounding temperature effects. While water temperatures were similar to blood temperatures during the colder sampling period (water: 26.3 ± 1.3°C; blood: 26.0 ± 1.09°C, mean ± SD), water temperatures were slightly higher than blood temperatures during the warmer sampling period (water: 29.1 ± 1.6°C; blood: 27.9 ± 1.4°C, mean ± SD). Similarly, *in vivo* Cuban dogfish temperatures were likely colder than surface water temperatures (28.5 ± 1.6; mean ± SD), as suggested by blood temperature readings (27.9 ± 1.9°C; mean ± SD). This could be due to thermal inertia, as these animals were hauled to the surface from capture temperatures of roughly 12°C. Further, due to the use of the insulating foam sleeve covering the vial of blood prior and during analysis for both species, as well as some daily temperature variation, samples may have been measured at a slightly different temperature than that at which the pH meter was calibrated. Thus, pH readings may be confounded by the internal pH meter temperature correction formula, which, in addition to the pH meter's buffer system, is designed for use on meat and dairy products and not for elasmobranch blood. It is recommended that future studies calibrate the pH meter and measure blood samples at temperatures as close to *in vivo* animal temperatures as possible to report true *in vivo* blood pH, which makes the measurement of blood pH in deepwater sharks considerably more difficult. If this is not feasible, we recommend measuring blood pH and calibrating the pH meter at the same temperature to reduce any possible errors and report relative changes in blood pH. Without these considerations, comparisons of blood pH between studies, even those focused on the same species, are limited.

Still, while conventional laboratory blood chemistry analysers are more accurate than field instruments, such as the i-STAT or the Hanna pH meter used here (Harrenstein *et al*., 2005; Gallagher *et al*., 2010; Harter *et al*., 2015), they are often impractical because they require the storage and transport of samples out of a field setting (Clark *et al*., 2011; Stoot *et al*., 2014). Additionally, analysing blood acid-base properties must occur within minutes of blood sampling to ensure accuracy (Stoot *et al*., 2014), making the use of field-worthy instruments a necessity. When sampling elasmobranch blood in particular, the requirements for an appropriate field analyser often include some water resistance, limited cost per sample, ease of use and low sensitivity to extreme temperatures. Given that the pH meter is waterproof, can be used repeatedly without single-use cartridges and can function in environments ranging −5 to 105°C (although samples cannot be analysed throughout this temperature range), it is a reasonable alternative to more commonly used pH analysers like the i-STAT system that require greater logistical considerations prior to use.

Expanding on the conservation physiology toolbox is of value to fisheries management, as blood chemistry metrics like pH can be used to predict lethal and sub-lethal endpoints and aid in species’ risk assessments (Gallagher *et al*., 2012). Given the current declines and the frequency of bycatch interactions experienced by many elasmobranch populations (Dulvy *et al*., 2014), understanding the drivers of stress during capture and predicting the consequences of capture for elasmobranchs is of great importance (Molina and Cooke, 2012). The pH meter, while limited in scope, can lower the cost of measuring relative changes in blood pH and increase the practicality of doing so in sub-tropical and tropical marine environments for at least two species of elasmobranchs if used appropriately.
